# Prior Knowledge, Older Age, and Higher Allowance Are Risk Factors for Self-Medication with Antibiotics among University Students in Southern China

**DOI:** 10.1371/journal.pone.0041314

**Published:** 2012-07-20

**Authors:** Hui Pan, Binglin Cui, Dangui Zhang, Jeremy Farrar, Frieda Law, William Ba-Thein

**Affiliations:** 1 Shantou-Oxford Clinical Research Unit, Shantou University Medical College, Shantou, Guangdong, P.R. China; 2 Oxford University Clinical Research Unit, Hospital for Tropical Diseases, Wellcome Trust Major Overseas Programme, Ho Chi Minh City, Viet Nam; 3 Consultant Office, Shantou University Medical College, Shantou, Guangdong, P.R. China; 4 Department of Microbiology and Immunology, Shantou University Medical College, Shantou, Guangdong, P.R. China; The University of Hong Kong, Hong Kong

## Abstract

**Background:**

Self-medication with antibiotics (SMA) has been reported among university students in many countries, but little research has been done on this issue in China. The objective of this study was to evaluate knowledge and behaviors of university students and risk factors concerning SMA.

**Methodology/Principal Findings:**

Using a novel questionnaire-based data collection instrument, an anonymous online survey was conducted with the students of Shantou University (STU), a university comprising 8 schools/colleges in eastern Guangdong, China. Of 1,300 respondents (13.8% of total eligible participants), 47.8% had self-treated with antibiotics. Logistic regression analysis identified prior knowledge of antibiotics (PKA), older age, and higher monthly allowance as independent risk factors for SMA. PKA significantly influenced students' knowledge about antibiotics, their uses, and common adverse reactions (all p<0.05). Among self-medicated students, 61.7% used antibiotics at least twice in the previous year. Community pharmacies were the major source of self-prescribed antibiotics. Reported common indications for SMA were sore throat (59.7%), fever (38.2%), cough (37.4%), runny nose (29.3%), and nasal congestion (28.7%). While 74.1% of self-medication episodes were based on students' own experiences, only 31.1% of students claimed to understand the package insert. Alteration of antibiotics and dosage during the course of self-treatment was made by 63.8% and 55.6% of students, respectively. At least two kinds of antibiotics were simultaneously taken by 82.6% of students. The majority of self-medicated students failed to complete the course of antibiotics. Adverse reactions were reported by 16.3% of students. Amoxicillin was the most common antibiotic used for self-medication.

**Conclusions:**

High prevalence of SMA was noted among STU students. Presence of risk factors and risk-associated behaviors/attitudes in the study population calls for focused educational intervention and stricter governmental legislation and regulation of antibiotic use and sale in pharmacies.

## Introduction

Irrational use or abuse of antibiotics is a global public health problem [1]. Antibiotic abuse not only leads to wastage of medical resources, but also contributes to the emergence of multi-drug resistant pathogens [2], [3]. While some developed countries have strict regulations on antibiotic use [4], developing countries are breeding grounds for drug-resistant bacteria due to poor supervision of antibiotic prescription [2], [5]. There is a risk of dissemination of resistant bacteria worldwide by bidirectional transmission between developed and developing countries [6].

Self-medication with antibiotics (SMA) is one form of antibiotic abuse, which is prevalent in developing countries with loose regulatory systems [7], [8]. The frequency of SMA ranges from 24% to 73.9% in Africa [9]–[11], 36.1% to 45.8% in the Middle East [12]–[14], 29% in South America [15], [16], 4% to 75% in Asia [1]. In comparison, lower SMA prevalence has been reported in developed countries with 3% in northern Europe, 6% in central Europe, and 19% in southern Europe [1].

Self-medication with antibiotics that are available over-the-counter (OTC) is a common practice in China. The self-reported SMA rates of outpatients vary from 28.7% to 62.5% [17], [18], with 59.4% in children (by parents) and youths [2] and 44.8% in general population [19]. Even higher rates (35.0%–90.4%) of SMA have been reported in Chinese university students [20], [21]. SMA in university students needs particular attention for the reasons that higher education level [4], [14], [22], younger age [4], [22], [23], and better social and economic background [14] are documented risk factors but perhaps also gives hope for modification of the practice through education.

In most medical schools/colleges in China, students receive a series of didactic lectures on antibiotics in the 2^nd^ year and have opportunities to observe antibiotic prescribing practice of senior physicians during their internship in the 4^th^ and 5^th^ years in the 5-year program or the 4^th^ to the 7^th^ years in the 7-year program. While antibiotic knowledge of medical students has been shown to be generally better than that of non-medical students in China [20], it is unknown how prior knowledge of antibiotics (PKA) could affect the behaviors or practice of students.

Despite high frequency of SMA in Chinese university students with apparent potential consequences, no research has been done on risk factors for SMA. Therefore, we conducted a cross-sectional survey to investigate antibiotic knowledge and SMA behaviors of university students so as to identify risk factors in a medium-sized city in southern China.

## Results

Out of 2,724 visits to our survey website, 1,300 STU students completed the online questionnaire. Nine non-student respondents were excluded from analysis. Tables 1, 2 and 3 show the demographic characteristics and relationships between SMA and classified groups of STU students, respectively. The mean age of students was 22.3 years (18–36±2.6).

**Table 1:** Demographic characteristics and self-medication with antibiotics (SMA) in STU students (n = 1,300).

Variable	Total students (n = 1300) n (%)	Self-medicated students (n = 621) n (%)	Odds ratio	95% confidence interval	p value
**Gender**					
Male	745 (57.3)	353 (47.4)	1	-	-
Female	555 (42.7)	268 (48.3)	1.04	(0.83–1.29)	0.75
**Age range (years)**					
18–19	103 (7.9)	31 (30.1)	1	-	-
20–21	484 (37.2)	178 (36.8)	1.35	(0.85–2.14)	0.20
22–23	402 (30.9)	181 (45.0)	1.90	(1.20–3.03)	<0.01
24–25	175 (13.5)	117 (66.9)	4.69	(2.77–7.93)	<0.0001
26–27	76 (5.8)	62 (81.6)	10.30	(5.02–21.06)	<0.0001
28–29	28 (2.2)	24 (85.7)	13.94	(4.46–43.54)	<0.0001
30–31	20 (1.5)	18 (90.0)	20.90	(4.57–95.61)	<0.0001
32–33	7 (0.5)	5 (71.4)	5.81	(1.07–31.56)	<0.05
34–36	5 (0.4)	5 (100.0)	Infinity	(NaN*-Infinity)	<0.01
**School/college**					
Medicine	634 (48.8)	375 (59.2)	1	-	-
Engineering	187 (14.4)	65 (34.8)	0.37	(0.26–0.52)	<0.0001
Science	92 (7.1)	31 (33.7)	0.35	(0.22–0.56)	<0.0001
Business	118 (9.1)	38 (32.2)	0.33	(0.22–0.50)	<0.0001
Law	90 (6.9)	41 (45.6)	0.58	(0.37–0.90)	0.01
Liberal Arts	77 (5.9)	25 (32.5)	0.33	(0.20–0.55)	<0.0001
Journalism and Communication	59 (4.5)	23 (39.0)	0.44	(0.26–0.76)	<0.01
Art and Design	43 (3.3)	23 (53.5)	0.79	(0.43–1.48)	0.47
**Education level**					
Undergraduate	933 (71.8)	379 (40.6)	1	-	-
Masters	343 (26.4)	221 (64.4)	2.65	(2.05–3.42)	<0.0001
Ph.D.	24 (1.9)	21 (87.5)	10.23	(3.03–34.55)	<0.0001

* NaN, not a number.

**Table 2:** Demographic characteristics and self-medication with antibiotics (SMA) in STU students (n = 1,300).

Variable	Total students (n = 1300) n (%)	Self-medicated students (n = 621) n (%)	Odds ratio	95% confidence interval	p value
**Allowance (in RMB/month)**					
≤500	548 (42.2)	215 (39.2)	1	-	-
500 to 1,000	668 (51.4)	352 (52.7)	1.73	(1.37–2.17)	<0.0001
1,001 to 2,000	74 (5.7)	46 (62.2)	2.54	(1.54–4.20)	<0.001
>2,000	10 (0.8)	8 (80.0)	6.20	(1.30–29.45)	0.01
**Health insurance over the past year** a					
None	64 (4.9)	30 (49.2)	1	-	-
Free medical care	124 (9.5)	55 (44.4)	0.82	(0.45–1.52)	0.54
College insurance	912 (70.2)	448 (49.1)	1.00	(0.59–1.68)	1
BMITS^b^	42 (3.2)	23 (54.8)	1.25	(0.57–2.75)	0.58
MIUTR^b^	379 (29.2)	181 (47.8)	0.94	(0.55–1.62)	0.84
Commercial health insurance	30 (2.3)	18 (60.0)	1.55	(0.64–3.76)	0.33
NRCOMI^b^	322 (24.8)	124 (38.5)	0.65	(0.37–1.12)	0.12
Others	17 (1.3)	7 (41.2)	0.72	(0.24–2.15)	0.56
**Hometown (province)**					
Guangdong	917 (70.5)	390 (42.5)	1	-	-
Shandong	51 (3.9)	46 (90.2)	12.43	(4.89–31.58)	<0.0001
Jiangxi	39 (3.0)	19 (48.7)	1.28	(0.68–2.44)	0.44
Hubei	35 (2.7)	16 (45.7)	1.14	(0.58–2.24)	0.71
Anhui	33 (2.5)	26 (78.8)	5.02	(2.16–11.68)	<0.0001
Zhejiang	32 (2.5)	18 (56.3)	1.74	(0.85–3.54)	0.12
Others	193 (14.9)	106 (54.9)	1.65	(1.20–2.25)	<0.01

a most of students had at least two kinds of health insurances;

b BMITS, basic medical insurance for town staff; MIUTR, medical insurance for urban and town residents; NRCOMI, new rural co-operative medical insurance.

**Table 3:** Classified groups of students and self-medication with antibiotics (n = 1,300).

Classified group of students	Total students (n = 1300) n (%)	Self-medicated students (n = 621) n (%)	Odds ratio	95% confidence interval	p value
Non-science group^a^	387 (29.8)	150 (38.8)	1	-	-
Science/non-medicine groupb, f, g	279 (21.5)	96 (34.4)	0.83	(0.60–1.14)	0.25
Medicine/non-PKA groupc, f, g	93 (7.2)	28 (30.1)	0.68	(0.42–1.11)	0.12
PKA groupd, g	541 (41.6)	347 (64.1)	2.83	(2.16–3.70)	<0.0001
Non-PKA group^e^	759 (58.4)	274(36.1)	1	-	-
PKA group^d^	541 (41.6)	347(64.1)	3.17	(2.52–3.98)	<0.0001

a students from schools/colleges of Liberal Arts, Law, Business, Art and Design, and Journalism and Communication;

b students from colleges of Science and Engineering;

c 1^st^-year medical students;

d all medical students except 1^st^ year students; PKA, prior knowledge of antibiotics;

e students from all groups except PKA group;

f p>0.05, science/non-medicine group vs. medicine/non-PKA group;

g p<0.0001, PKA group vs. science/non-medicine group; p<0.0001, PKA group vs. medicine/non-PKA group.

### Prevalence of SMA and factors associated with SMA

Of 1,300 respondents, 621 (47.8%) had self-treated with antibiotics. As shown in Tables 1 and 2, school/college, education level, allowance per month, and hometown (viz. Shandong and Anhui provinces) were significantly associated with SMA (all p≤0.01). There were significant differences in SMA rate between medical students and the students from other disciplines (all p≤0.01), except the School of Art and Design (p>0.05). Specifically, SMA frequency was significantly higher in the PKA group than that in the other groups (all p<0.0001, Table 3).

To identify risk factors associated with SMA, we first analyzed the relationship between different SMA-associated factors by bivariate correlation analysis. We included only the variables significantly and independently associated with SMA, as shown by Chi-square test and correlation analysis, in a logistic regression model, where SMA was used as dependent variable, and real age, allowance, and PKA as independent variables. Table 4 shows that older age, higher allowance, and PKA were risk factors for SMA (all p<0.01).

**Table 4:** Logistic regression analysis of SMA-associated factors in STU students (n = 1,300).

Explanatory variable	p value	OR	95% CI
**Age**	<0.0001	1.23	(1.16–1.30)
**Allowance (in RMB/month)**			
≤500	Reference	-	-
500 to 1,000	<0.01	1.49	(1.17–1.91)
>1,000	<0.01	2.18	(1.29–3.68)
**Prior knowledge of antibiotics (PKA)**	<0.001	2.23	(1.74–2.87)

SMA, self-medication with antibiotics.

Since PKA was most significantly associated with SMA (OR: 2.23, Table 4), we scrutinized the influence of PKA by comparing self-medicated and non-self-medicated students with respect to their knowledge about antibiotics and SMA behaviors (Table 5), and the findings are as described below.

**Table 5:** Knowledge and misconception about antibiotics among STU students (n = 1300).

	n (%) of YES answer in non-SMA group^a^			n (%) of YES answer in SMA group^a^		
Question	Total (n = 679)	Non-PKA^b^ (n = 485)	PKA^b^ (n = 194)	Total (n = 621)	Non-PKA^b^ (n = 274)	PKA^b^ (n = 347)
Do you know what antibiotics are?^c^	528 (77.8)^d^	346 (71.3)e, g	182 (93.8)f, g	559 (90.0)^d^	220 (80.3)e, h	339 (97.7)f, h
Antibiotics are used for viral infections	232 (43.9)^d^	174 (50.3)^g^	58 (31.9)f, g	181 (32.4)^d^	114 (51.8)^h^	67 (19.8)f, h
Antibiotics are used for bacterial infections	495 (93.8)	316 (91.3)^g^	179 (98.4)^g^	524 (93.7)	191 (86.8)^h^	333 (98.2)^h^
Broad-spectrum antibiotics are better than narrow-spectrum antibiotics	72 (13.6)	60 (17.3)^g^	12 (6.6)^g^	66 (11.8)	30 (13.6)	36 (10.6)
Higher doses result in faster recovery	44 (8.3)	28 (8.1)	16 (8.8)	45 (8.1)	21 (9.6)	24 (7.1)
Lower doses result in less adverse reactions	274 (51.9)^d^	194 (56.1)e, g	80 (44.0)^g^	239 (42.8)^d^	102 (46.4)^e^	137 (40.4)
Switching antibiotics enhances drug effects	267 (50.6)^d^	192 (55.5)e, g	75 (41.2)f, g	149 (26.7)^d^	77 (35.0)e, h	72 (21.2)f, h
Switching antibiotics reduces adverse reactions	122 (23.1)	83 (24.0)	39 (21.4)	108 (19.3)	41 (18.6)	67 (19.8)
Intravenous is better than oral medication	273 (51.7)^d^	212 (61.3)e, g	61 (33.5)^g^	218 (39.0)^d^	102 (46.4)e, h	116 (34.2)^h^
Common adverse reactions of antibiotics are:						
Nausea	445 (84.3)	284 (82.1)	161 (88.5)	472 (84.4)	178 (80.9)	294 (86.7)
Vomiting	423 (80.1)^d^	267 (77.2)e, g	156 (85.7)^g^	420 (75.1)^d^	145 (65.9)e, h	275 (81.1)^h^
Diarrhea	346 (65.5)	204 (59.0)^g^	142 (78.0)^g^	374 (66.9)	129 (58.6)^h^	245 (72.3)^h^
Rash	400 (75.8)	238 (68.8)e, g	162 (89.0)f, g	401 (71.7)	128 (58.2)e, h	273 (80.5)f, h
Vaginal thrush	113 (21.4)^d^	45 (13.0)^g^	68 (37.4)^g^	179 (32.0)^d^	36 (16.4)^h^	143 (42.2)^h^
Drug resistance	370 (70.1)^d^	207 (59.8)e, g	163 (89.6)^g^	450 (80.5)^d^	158 (71.8)e, h	292 (86.1)^h^

a non-SMA, non-self-medicated students; SMA, self-medicated students;

b non-PKA, without prior knowledge of antibiotics; PKA, with prior knowledge of antibiotics;

c students who answered “NO” were not allowed to answer other questions;

d p<0.05, non-SMA vs. SMA;

e p<0.05, non-SMA/non-PKA vs. SMA/non-PKA;

f p<0.05, non-SMA/PKA vs. SMA/PKA;

g p<0.05, non-SMA/non-PKA vs. non-SMA/PKA;

h p<0.05, SMA/non-PKA vs. SMA/PKA.

### Knowledge and misconceptions about antibiotics and their uses

In general, the SMA group had better knowledge than the non-SMA group, and the PKA group was also better than the non-PKA group in the knowledge of antibiotics and their uses (Table 5). For example, lower proportion of the PKA group thought that antibiotics are suitable for viral infections, switching antibiotics would enhance drug effects, and intravenous is more effective than oral medication (all p<0.05, Table 5). The PKA group was also significantly better than the non-PKA group in the knowledge of common adverse reactions of antibiotics, such as vomiting, diarrhea, rash, vaginal thrush, and emergence of drug resistance (all p<0.05, Table 5).

### SMA behaviors and attitudes

Among 621 students with an SMA history, 61.7% (373/605, 16 incomplete data) of students had self-treated with antibiotics at least twice in the past one year, with the maximal frequency of 20; and there was no difference between the PKA and non-PKA groups (60.8% vs. 62.8%, p = 0.61). Some (39.6%) of the self-medicated students had taken the same antibiotics with different brand names simultaneously, and 82.6% (497/602, 19 incomplete data) of students self-treated with at least 2 kinds of antibiotics during a single illness. There was no difference between the PKA and non-PKA groups regarding the number of antibiotics used during a single illness.

Adverse reactions, including nausea, vomiting, diarrhea, dizziness, tinnitus, somnolence, muscle soreness, and rash, were reported by 101 (16.3%) self-medicated students. In response to the adverse reactions, 77.6% stopped taking antibiotics, 38.8% consulted a doctor, 23.5% switched to another antibiotic, 17.4% consulted pharmacy staff, 13.3% consulted family members or friends, and 11.2% took no action. Higher rate of self-reported adverse reactions was observed in the PKA group, as compared to that in the non-PKA group (19.0% vs. 12.8%, p<0.05).

Other behaviors and attitudes of SMA among self-medicated STU students are shown in Table S1. Top 10 antibiotics that students had used for self-medication are summarized in Table 6. The most commonly used antibiotic was amoxicillin.

**Table 6:** Ten most commonly used antibiotics for self-medication in STU students (n = 508*).

Class of antibiotics	Name of antibiotics	n (%)
Penicillins	Amoxicillin	246 (48.4)
	Penicillin	93 (18.3)
Cephalosporins (1st-generation)	Cephradine	99 (19.5)
	Cephalexin	30 (5.9)
Macrolides	Roxithromycin	64 (12.6)
	Erythromycin	56 (11.0)
	Azithromycin	27 (5.3)
Quinolones	Norfloxacin	41 (8.1)
	Levofloxacin	39 (7.7)
Others	Chloramphenicol	28 (5.5)

* 69 out of 621 self-medicated students failed to mention the names of antibiotics they used.

## Discussion

High prevalence of SMA has been documented among university students in developing countries including China. SMA rates among Chinese university students were 38% (178/468) in northwest China (Gansu province) [20], 90% (452/500) in Beijing [21], and 48% (621/1300) in this study in southern China. In African countries, SMA prevalence has been reported as 24% (169/706) with female Nigerian university students [9] and 76% (852/1121) with Sudanese university students [24]. These findings suggest that SMA-associated consequences, such as adverse reactions, drug resistance, and treatment failure [8], are predictably imminent in China and other developing countries.

### SMA risk factors

Risk factors for SMA have been described as higher education level in European, Jordanian, and Sudanese adults [4], [11], [14], better knowledge about antimicrobials in the British community [22], lower education level and non-science major in Nigerian university students [9], younger age in Europe and Viet Nam [4], [22], [23], adults aged 18–39 and higher income in the Jordanian community [14], adults aged 40–59 and lower income in the Sudanese community [11], and being female in the Sudanese and British communities [11], [22]. This study, however, identified PKA, older age, and higher allowance as independent risk factors for SMA.

#### Prior knowledge

The observed significantly higher rate of SMA in medical students or better SMA-related knowledge in the SMA group were contributed by the PKA group that represents senior medical students who had taken formal lectures about antibiotics. Their PKA probably has led to a false sense of confidence in self-diagnosis and self-management. Alternatively, medication-related knowledge plus easy access to purchasing antibiotics without prescription under the loose regulatory system might have encouraged their SMA practice. Influence of PKA on students' knowledge has been described previously [20], [25], but this study is the first one to report the influence of PKA on SMA behaviors.

#### Age

The rationale behind higher SMA rate in older students could be cumulative illness episodes and/or prior experience of SMA, which contribute to SMA.

#### Allowance

Lower SMA rate was recorded in the students with the monthly allowance of less than 500 RMB in this study. As the average living cost for a Shantou citizen was 1,101 RMB per month according to Annual Statistic Report of Shantou in 2011 issued by Statistics Department of Shantou government (available at: http://sttj.shantou.gov.cn/tjsj/tjnj2011/1/_.htm), the students with monthly allowance of less than 500 RMB could have either visited the school health center that offers free healthcare, or tolerated minor illnesses such as colds. SMA could be one option for them to save time and hassle especially when they were busy during examination season. Nonetheless, no significant difference in antibiotic knowledge or SMA behaviors was observed among students with different allowances except for SMA rate (data not shown).

#### Other SMA-associated factors

Although higher SMA rates were observed with the students from Shandong and Anhui provinces, hometown was not an independent risk factor in this study. It could be explained by the fact that drugs including antibiotics can be purchased freely in community pharmacies and traditional Chinese medicine (TCM) pharmacies throughout China. The hometown difference we observed is in fact due to that these students were older, had better allowance, and/or had higher education level (data not shown). Higher SMA rates with medical major and postgraduate education in this study are also contributed by PKA. Therefore, risk factors for SMA appear to vary with country, social and economic background, and study population.

### Misconceptions and misbeliefs

Misconceptions and misbeliefs concerning antibiotics observed herein have been documented in several Chinese studies [13], [21], [26]. For example, antibiotic use for common cold was reported in 89.6% of university students in Beijing [21]. In addition, intravenous was described as better than oral medication by 26.9% of Chinese parents of children in a previous study [26] and also by 39% (218/559) of our students. This misconception might have come from their experience with the routine practice of Chinese doctors to administer intravenous preferentially over oral medications from the pressure of demanding patients and for high financial incentives [2], [19], [27].

Focused education on antibiotics and its use, which is integrated in medical education, had a positive influence on the knowledge among the students in this study. PKA seemed to have helped most students (65.8%–78.8%) in the PKA group dispel some misconceptions, such as switching antibiotics for enhanced drug effects and preference of intravenous treatment. Similar positive impact of PKA was reported in the studies in Gansu and Zhejiang provinces of China [20], [25].

### SMA behaviors

PKA, however, had just minor positive influence on students' behaviors towards SMA with respect to frequency, indication, frequency of switching type and dosage of antibiotics, and consumption of multiple antibiotics. For example, the majority of both PKA and non-PKA groups (60.8% vs. 62.8%, p = 0.61) self-medicated at least twice in the past one year. Our findings are supported by a study in Turkey that points out that antibiotic knowledge is not always compatible with behaviors [13].

The most common reasons for SMA were convenience and cost-saving in this study. Similar reasons have been reported in the studies with children in Mongolia and Viet Nam [7], [8]. The reason could be that non-prescribed antibiotics can be purchased conveniently and affordably in community pharmacies in China and other developing countries [7], [20].

Antibiotics have been used mostly for common cold [7] and diarrhea [2], which are considered as low-severity illnesses and self-treatable by respondents in previous studies [2], [7]. Likewise, STU students usually self-medicated for symptoms of respiratory tract infections. Of note, the possibility of influence on SMA rates by the influenza outbreak before or during this study is not supported by our analysis (data not shown).

Majority of STU students were at risk by making decision to use antibiotics by themselves (particularly the PKA group) or by relying on salespersons in pharmacies and family members. The respondents in other studies also reported the same [7], [8]. Both family members and often the salespersons in pharmacies have inadequate knowledge about antibiotics [2], [7], and the latter make their profits out of the recommendations they make on certain medicines, despite these recommendations not being appropriate in many circumstances [2]. The non-PKA group was at even greater risk of adverse reactions, drug resistance, and treatment failure, because the majority either decided the type of antibiotics and dosage by themselves or poorly understood the instruction in the package insert.

Other risky self-reported behaviors STU students engaged in include alteration of antibiotics and the dosage during the course of self-treatment by more than 55% of students and taking multiple antibiotics or similar antibiotics with various names simultaneously by about 40% of students. Switching antibiotics over a very short time would be the equivalent to the use of multiple antibiotics and incomplete courses. Frequent administration of antibiotics in small doses for short period can also cause a selective pressure on bacteria [8]. Poor compliance with self-medication we observed in this study has as well been reported in other studies with rural population in Greece and children in Vietnamese and Mongolian communities [7], [8], [28].

We also noted hazardous attitudes in STU students. Despite the fact that China is one of the largest producers and consumers of counterfeit drugs [29], the majority of self-medicated students cared little about counterfeit antibiotics. Also, while lacking confidence in self-treating common infectious diseases successfully, more than 90% of the self-medicated students, especially in the non-PKA group, still regarded SMA as an acceptable practice.

### Consequences of SMA

#### Adverse reactions

Adverse reactions caused by antibiotics are an important problem. According to the report of the State Food and Drug Administration (SFDA) of China in 2009, antimicrobials are responsible for 55.2% of all reported adverse reactions from medication. However, a limited number of studies worldwide have reported only up to11% of cases with adverse reactions regarding SMA [1]. In comparison, the occurrence in this study was higher (around 17%), which could be due to simultaneous consumption of multiple antibiotics, incorrect dosage, and/or consumption of counterfeit/substandard antibiotics. Adverse reactions were also the cause of poor compliance and switching of antibiotics by STU students.

#### Drug resistance

The top 10 antibiotics used for SMA in this study represent the commonly abused antibiotics for self-medication in Greece [28], Nigeria [9], Mongolia [7], Viet Nam [8], Indonesia [30], and China [18]. In general, penicillins (especially amoxicillin) and cephalosporins are the most commonly abused antibiotics in these countries.

It has been shown that drug resistance is directly proportional to non-prescription use of antibiotics [1], [8]. Although we could not establish a causal link between SMA behaviors and drug resistance in this study, the high prevalence of SMA, limited knowledge of appropriate antibiotic use, and poor behaviors and attitudes towards SMA among STU students could contribute to the pressure on bacteria and the development of drug resistance.

SMA is a global problem [1]. While some SMA-related behaviors such as reasons for self-medication, indication of use, and poor compliance described herein are similar to those reported in other countries with loose regulatory systems [7], [8], [28], there are certain issues unique to China, for example, crowded conditions in clinical facilities, long waiting hours, very short consultation time, lack of privacy, high cost at hospitals, and lack of trust in healthcare professionals [31]–[33]. Without improved healthcare system, these issues would discourage people to seek professional medical help.

There are limitations in this study. While SUMC is the largest college in STU and thus likely contributed to higher proportion of survey respondents, the online health survey also could have attracted medical students or students who were more health-conscious, which could lead to opportunistic sampling to some extent. Since the student responses were self-reported, subjective inaccuracies should be taken into consideration in interpreting our results. Another limitation is that prevalence rate and risk factors in general population could be different from those in university students. As any intervention should be based on the risk factors present in study population, similar survey should be done in non-university-student population using our survey instrument, which though designed for Chinese university students can also be applicable to general population and even in any country where SMA is prevalent.

In summary, this study demonstrates that SMA is prevalent in STU students and that PKA, older age, and higher monthly allowance are independent risk factors for SMA. Given that multiple risk factors and risk-associated behaviors/attitudes are present in the study population, who are future community leaders and role models, focused education through seminars, workshops, and social media on rational use of antibiotics with an emphasis on misconceptions, risks, and consequences associated with self-medication is recommended for university students. Since SMA is a national problem, to protect general population against foreseeable threats, implementation of stricter governmental legislation and regulation on sale and use of non-prescribed medicines including antibiotics in pharmacies is urgently needed.

## Methods

### Study population

This survey was carried out during April-May, 2011 in Shantou University (STU) located in Shantou, eastern Guangdong province, southern China. STU comprises 8 schools/colleges (Table 1), with the Medical College (Shantou University Medical College, SUMC) being the largest and its students accounting for 25.7% of total enrolled students at STU (9,398) in 2011.

### Questionnaire

A structured questionnaire was designed as a novel data collection instrument based on previous studies [8], [9], [13], [20], and consisted of three parts with 24 questions regarding SMA behaviors in part A, 4 questions related to knowledge and functions of antibiotics and self-medication in part B, and 8 questions on demographic information in part C. Questions included closed-ended (yes/no, single choice, and multiple choice), matrix single-choice, and open-ended questions (File S1). SMA was defined as taking OTC antibiotics (without prescription) for self-treatment.

The questionnaire was drafted in English, translated into Chinese, and back translated into English to assure the accuracy in translation, and reviewed by clinicians, epidemiologist, microbiologist, and medical and non-medical students for validation. A pilot test was performed with a group of university students.

### Administration of online survey

Participants were invited via the STU website to take an anonymous online survey (available at: http://www.sojump.com/jq/695790.aspx [Chinese version] and http://www.sojump.com/jq/757672.aspx [English version]). Posters, flyers, and financial incentives were used to enhance response rate. To prevent multiple submissions, only one IP address per submission was allowed. All STU students were eligible to participate in the study, whereas non-STU students were excluded.

### Ethics and confidentiality

Ethical approval was obtained from SUMC. Security and confidentiality of all the submitted questionnaires were guaranteed by the online survey website.

### Data analysis

Only completed questionnaires from STU students were collected automatically by the survey software and downloaded into a Microsoft Excel sheet for analysis with SPSS (PASW) 17.0. Chi-square test was used for comparison between variables, bivariate correlation and logistic regression was carried out to evaluate associations between SMA and the characteristics of respondents.

To evaluate risk factors for SMA, the respondents were divided into PKA group (including all medical students except the 1^st^ year who would only have received anatomy and no other medically related subjects, n = 541) and non-PKA group (including the 1^st^ year medical students and all non-medical students, n = 759). The non-PKA group was subdivided into non-science group (including students from schools/colleges of Liberal Arts, Law, Business, Art and Design, and Journalism and Communication, n = 387), science/non-medicine group (including students from colleges of Science and Engineering, n = 279), and medicine/non-PKA group (i.e., the 1^st^-year medical students, n = 93).

## Supporting Information

Table S1 SMA behaviors and attitudes of self-medicated students.
(DOC)

File S1 Questionnaire for self-medication with antibiotics.
(PDF)
