# Supplementary material for: Prior Knowledge, Older Age, and Higher Allowance Are Risk Factors for Self-Medication with Antibiotics among University Students in Southern China
Source: PLoS One. 2012 Jul 20;7(7):e41314. doi: 10.1371/journal.pone.0041314 (PMC3401104; doi:10.1371/journal.pone.0041314)
Supplement: Table S1 — SMA behaviors and attitudes of self-medicated students. (DOC) [file pone.0041314.s001.doc]

**Table S1. SMA behaviors and attitudes of self-medicated students (n=621)**

|  | **n (%) of YES answer** | | |  |
| --- | --- | --- | --- | --- |
| **Question** | **Total (n=621)** | **Non-PKA^a^(n=274)** | **PKA^a^ (n=347)** | **p value^b^** |
| 1. **Reasons of SMA (MC^c^)** |  |  |  |  |
| Convenience | 546 (87.9) | 257 (93.8) | 289 (83.3) | <0.0001 |
| Cost saving | 277 (44.6) | 130 (47.5) | 147 (42.4) | 0.21 |
| Lack of trust in prescribing doctor | 44 (7.1) | 25 (9.1) | 19 (5.5) | 0.08 |
| 1. **Main source of antibiotics for SMA (MC^c^)** |  |  |  |  |
| Community pharmacies | 582 (93.7) | 256 (93.4) | 326 (94.0) | 0.79 |
| Leftover from previous prescription | 231 (37.2) | 118 (43.1) | 113 (32.6) | <0.01 |
| Traditional Chinese Medicine practitioners | 45 (7.3) | 32 (11.7) | 13 (3.8) | <0.001 |
| 1. **Indications for SMA (MC^c^)** |  |  |  |  |
| Sore throat | 371 (59.7) | 169 (61.7) | 202 (58.2) | 0.38 |
| Fever | 237 (38.2) | 91 (33.2) | 146 (42.1) | <0.05 |
| Cough | 232 (37.4) | 93 (33.9) | 139 (40.1) | 0.11 |
| Runny nose | 182 (29.3) | 72 (26.3) | 110 (31.7) | 0.14 |
| Nasal congestion | 178 (28.7) | 83 (30.3) | 95 (27.4) | 0.43 |
| Diarrhea | 107 (17.2) | 40 (14.6) | 67 (19.3) | 0.12 |
| Skin wounds | 101 (16.3) | 60 (21.9) | 41 (11.8) | <0.001 |
| Aches and pains | 76 (12.2) | 47 (17.2) | 29 (8.4) | <0.001 |
| Vomiting | 30 (4.8) | 11 (4.0) | 19 (5.5) | 0.39 |
| 1. **Knowledge of how to self-medicate (MC^c^)** |  |  |  |  |
| My own experience | 460 (74.1) | 172 (62.8) | 288 (83.0) | <0.0001 |
| Previous doctor’s prescription | 218 (35.1) | 108 (39.4) | 110 (31.7) | <0.05 |
| Opinion of family members | 195 (31.4) | 135 (49.3) | 60 (17.3) | <0.0001 |
| Recommendation by community pharmacists | 175 (28.2) | 106 (38.7) | 69 (19.9) | <0.0001 |
| 1. **Choice of antibiotics was based on (MC^c^)** |  |  |  |  |
| Indications for use | 514 (82.8) | 217 (79.2) | 297 (85.6) | <0.05 |
| Type of antibiotics | 391 (63.0) | 146 (53.3) | 245 (70.6) | <0.0001 |
| Adverse reactions | 371 (59.7) | 158 (57.7) | 213 (61.4) | 0.35 |
| Brand | 264 (42.5) | 141 (51.5) | 123 (35.5) | <0.0001 |
| Cost | 229 (36.9) | 79 (28.8) | 150 (43.2) | <0.001 |
| 1. **Review of package insert** |  |  |  |  |
| Always | 482 (77.6) | 202 (73.7) | 280 (80.7) | <0.05 |
| Sometimes | 130 (20.9) | 68 (24.8) | 62 (17.9) | <0.05 |
| Never | 9 (1.5) | 4 (1.5) | 5 (1.4) | 0.98 |
| 1. **Understanding package insert (non-PKA: n=** **270; PKA: n=341^d^)** | | | | |
| Partly understood | 413 (67.6) | 230 (85.2) | 183 (53.7) | <0.0001 |
| Fully understood | 190 (31.1) | 34 (12.6) | 156 (45.8) | <0.0001 |
| Did not understand at all | 8 (1.3) | 6 (2.2) | 2 (0.6) | 0.08 |
| 1. **Determination of dosage (MC^c^)** | | | | |
| By checking the package insert | 587 (94.5) | 259 (94.5) | 328 (94.5) | 1 |
| By own previous experience | 232 (37.4) | 99 (36.1) | 133 (38.3) | 0.57 |
| By consulting a doctor | 175 (28.2) | 82 (29.9) | 93 (26.8) | 0.39 |
| By consulting a pharmacist | 137 (22.1) | 87 (31.8) | 50 (14.4) | <0.0001 |
| By consulting family members/friends | 106 (17.1) | 77 (28.1) | 29 (8.4) | <0.0001 |
| By guessing | 73 (11.8) | 35 (12.8) | 38 (11.0) | 0.48 |
| 1. **Change of dosage during the course of self-treatment** | | | | |
| Sometimes | 318 (51.2) | 148 (54.0) | 170 (49.0) | 0.21 |
| Never | 276 (44.4) | 116 (42.3) | 160 (46.1) | 0.35 |
| Always | 27 (4.4) | 10 (3.7) | 17 (4.9) | 0.45 |
| 1. **Reasons for changing the dosage (MC^c^, non-PKA: n=** **158; PKA: n=187)** | | | | |
| Improving conditions | 181 (52.5) | 86 (54.4) | 95 (50.8) | 0.5 |
| Worsening conditions | 166 (48.1) | 78 (49.4) | 88 (47.1) | 0.67 |
| To reduce adverse reactions | 117 (33.9) | 56 (35.4) | 61 (32.6) | 0.58 |
| Drug insufficient for complete treatment | 19 (5.5) | 9 (5.7) | 10 (5.4) | 0.89 |
| 1. **Switching antibiotics during the course of self-treatment** | | | | |
| Sometimes | 372 (59.9) | 162 (59.1) | 210 (60.5) | 0.72 |
| Never | 225 (36.2) | 102 (37.2) | 123 (35.5) | 0.65 |
| Always | 24 (3.9) | 10 (3.7) | 14 (4.0) | 0.8 |
| 1. **Reasons for switching antibiotics (MC^c^, non-PKA: n=** **172; PKA: n=224)** | | | | |
| Ineffectiveness | 276 (69.7) | 107 (62.2) | 169 (75.5) | <0.01 |
| Insufficient drug | 146 (36.9) | 77 (44.8) | 69 (30.8) | <0.01 |
| Adverse reactions | 90 (22.7) | 41 (23.8) | 49 (21.9) | 0.64 |
| High cost | 34 (8.6) | 20 (11.6) | 14 (6.3) | 0.06 |
| 1. **Timing of discontinuation of antibiotics (MC^c^)** | | | | |
| After symptoms disappeared | 455 (73.3) | 222 (81.0) | 233 (67.2) | <0.001 |
| At the completion of the course | 176 (28.3) | 67 (24.5) | 109 (31.4) | 0.06 |
| A few days after the recovery | 88 (14.2) | 22 (8.0) | 66 (19.0) | <0.0001 |
| After consulting a doctor or pharmacist | 85 (13.7) | 39 (14.2) | 46 (13.3) | 0.73 |
| After use up of antibiotics | 76 (12.2) | 39 (14.2) | 37 (10.7) | 0.18 |
| After a few days regardless of the outcome | 72 (11.6) | 36 (13.1) | 36 (10.4) | 0.29 |
| 1. **Concern about unintended consumption of counterfeit antibiotics** | | | | |
| Yes | 248 (39.9) | 123 (44.9) | 125 (36.0) | <0.05 |
| 1. **Attitude towards self-medication as personal healthcare** | | | | |
| Good or acceptable practice | 559 (90.0) | 255 (93.1) | 304 (87.6) | <0.05 |
| 1. **Confidence in self-treating common infectious diseases with antibiotics** | | | | |
| Successfully treatable | 236 (38.0) | 85 (31.0) | 151 (43.5) | <0.01 |

^a^ non-PKA, without prior knowledge of antibiotics; PKA, with prior knowledge of antibiotics; ^b^ non-PKA group vs. PKA group; ^c^ MC, multiple choice; ^d^ one incomplete data in PKA group (1/342)
