# Supplementary material for: Prior Knowledge, Older Age, and Higher Allowance Are Risk Factors for Self-Medication with Antibiotics among University Students in Southern China
Source: PLoS One. 2012 Jul 20;7(7):e41314. doi: 10.1371/journal.pone.0041314 (PMC3401104; doi:10.1371/journal.pone.0041314)
Supplement: File S1 — Questionnaire for self-medication with antibiotics. (PDF) [file pone.0041314.s002.pdf]

File S1: Questionnaire for self-medication with antibiotics

Part A: Self-medication behaviors

1	**Have you ever taken antibiotics?**<br>A. Yes<br>B. No<br>*If **NO**, please go to Part B Question 1*
2	**Have you ever treated yourself (self-medicated) with antibiotics?**<br>A. Yes<br>B. No<br>*If **NO**, please go to Part B Question 1*
3	**How many times did you treat yourself with antibiotics in the past one year?**<br>____________________
4	**What was (were) your reason(s) of self-medication with antibiotics?** ***(check more than one if applicable)***<br>A. Cost saving<br>B. Convenience<br>C. Lack of trust in prescribing doctor<br>D. Others (specify)____________________
5	**For which of the following complaint(s) did you use antibiotics?** ***(check more than one if applicable)***<br>A. Runny nose<br>B. Nasal congestion<br>C. Cough<br>D. Sore throat<br>E. Fever<br>F. Aches and pains<br>G. Vomiting<br>H. Diarrhea<br>I. Skin wounds<br>J. Others (specify)____________________
6	**Your selection of antibiotics was based on…** ***(check more than one if applicable)***<br>A. Recommendation by community pharmacists<br>B. Opinion of family members<br>C. Opinion of friends<br>D. My own experience<br>E. Recommendation by net citizens<br>F. Previous doctor’s prescription<br>G. The advertisement
7	**What did you consider when selecting antibiotics?** ***(check more than one if applicable)***<br>A. Type of antibiotics<br>B. Brand of antibiotics<br>C. Price of antibiotics<br>D. Indications for use<br>E. Adverse reactions<br>F. Others (specify)____________________
8	**Where did you usually obtain antibiotics from for self-medication?** ***(check more than one if applicable)***<br>A. Community pharmacies<br>B. TCM practitioners

	C. Leftover from previous prescription<br>D. Online shopping/E-pharmacies<br>E. Others (specify)______________________________
9	**Did you ever check the instructions come with the package insert of antibiotics for self-treatment?**<br>A. Yes, always<br>B. Yes, sometimes<br>C. Never<br>*If **Never**, please go to Question 11*
10	**How much did you understand the instructions?**<br>A. Fully understood<br>B. Partly understood (percentage:________)<br>C. Did not understand at all
11	**How did you know the dosage of antibiotics? *(check more than one if applicable)***<br>A. By checking the package insert<br>B. By consulting a doctor<br>C. By consulting a pharmacist<br>D. By consulting family members/friends<br>E. From the newspapers, magazines, books, or TV programs<br>F. From the Internet<br>G. From my previous experience<br>H. By guessing the dosage by myself
12	**Did you ever change the dosage of antibiotics deliberately during the course of self-treatment?**<br>A. Yes, always<br>B. Yes, sometimes<br>C. Never<br>*If **Never**, please go to Question 14*
13	**Why did you change the dosage of antibiotics during the course of self-treatment? *(check more than one if applicable)***<br>A. Improving conditions<br>B. Worsening conditions<br>C. To reduce adverse reactions<br>D. Drug insufficient for complete treatment<br>E. Others (specify)______________________________
14	**Did you ever switch antibiotics during the course of self-treatment?**<br>A. Yes, always<br>B. Yes, sometimes<br>C. Never<br>*If **Never**, please go to Question 16*
15	**Why did you switch antibiotics during the course of self-treatment? *(check more than one if applicable)***<br>A. The former antibiotics did not work<br>B. The former antibiotics ran out<br>C. The latter one was cheaper<br>D. To reduce adverse reactions<br>E. Others (specify)______________________________
16	**How many different antibiotics did you take maximally during a single illness?**<br>______________________________
17	**Are you concerned that you might have taken counterfeit antibiotics?**<br>A. Yes, very much<br>B. Yes, somewhat

	C. No
18	**Have you ever found out that you had taken the same antibiotics with different names at the same time?**<br>A. Yes<br>B. No
19	**When did you normally stop taking antibiotics?** ***(check more than one if applicable)***<br>A. After a few days regardless of the outcome<br>B. After symptoms disappeared<br>C. A few days after the recovery<br>D. After antibiotics ran out<br>E. At the completion of the course<br>F. After consulting a doctor/pharmacist<br>G. Others (specify)____________________
20	**Have you ever had any adverse reaction when you took antibiotics for self-medication?**<br>A. Yes (specify)____________________<br>B. No<br>*If **NO**, please go to Question 22*
21	**What did you do for the adverse reactions?** ***(check more than one if applicable)***<br>A. Stopped taking antibiotics<br>B. Switched to another antibiotic<br>C. Consulted pharmacy staff<br>D. Consulted a doctor<br>E. Consulted family members/friends<br>F. Nothing<br>G. Others (specify)____________________
22	**Please write down the names of antibiotics you have ever taken for SELF-MEDICATION:**<br>A. __________ B.__________ C.__________<br>D. __________ E.__________
23	**What do you think about self-medication with antibiotics for self health care?**<br>A. Good practice<br>B. Acceptable practice<br>C. Not acceptable practice
24	**Do you think you can treat common infectious diseases with antibiotics successfully by yourself?**<br>A. Yes, I can<br>B. Not sure<br>C. No, I cannot

Part B: Knowledge

1	**Do you know what are antibiotics?**<br>A. Yes<br>B. No
2	**What are antibiotics used for?** ***(check more than one if applicable)***<br>A. Virus infection<br>B. Bacterial infection<br>C. Others (specify)____________________
3	**Which of the following statement(s) about antibiotics is (are) correct?** ***(True/False)***<br>A. Broad-spectrum antibiotics are better than narrow-spectrum ones<br>B. Higher doses result in faster recovery

	C. Lower doses result in less adverse reactions<br>D. Switching antibiotics enhances drug effects<br>E. Switching antibiotics reduces adverse reactions<br>F. Intravenous is better than oral medication
4	**What is (are) the common adverse reaction(s) of antibiotics?** ***(check more than one if applicable)***<br>A. Nausea<br>B. Vomiting<br>C. Diarrhea<br>D. Rash<br>E. Vaginal thrush<br>F. Drug resistance

Background

**Age (years):** ________	**Gender:** A. Male B. Female
**School/college:**<br>A. College of Science<br>B. Medical College<br>C. College of Liberal Arts<br>D. College of Engineering<br>E. Law School<br>F. Business School<br>G. Cheung Kong School of Art and Design<br>H. Cheung Kong School of Journalism and Communication	
**Program:**<br>A. Undergraduate<br>B. Masters<br>C. Ph.D.	
**Year of your study: (e.g., 2nd year)**<br>______________________________	
**Your monthly allowance:**<br>A. ≤500 RMB<br>B. 500 to 1,000 RMB<br>C. 1,001 to 2,000 RMB<br>D. >2,000 RMB	
**What kind(s) of health insurance(s) do you have this year?** ***(check more than one if applicable)***<br>A. Free medical care<br>B. College insurance<br>C. Basic medical insurance for town staff<br>D. Medical insurance for urban and town residents<br>E. Commercial health insurance<br>F. New rural co-operative medical insurance<br>G. None<br>H. Others______________________	
**Hometown:** __________Province________City	

*** The end ***
